# Supplementary material for: Ligand-Dependent Conformational Transitions in Molecular Dynamics Trajectories of GPCRs Revealed by a New Machine Learning Rare Event Detection Protocol
Source: Molecules. 2021 May 20;26(10):3059. doi: 10.3390/molecules26103059 (PMC8161244; doi:10.3390/molecules26103059)
Supplement: Supplementary file 1 [file molecules-26-03059-s001.zip › molecules-1206538-supplementary.pdf]

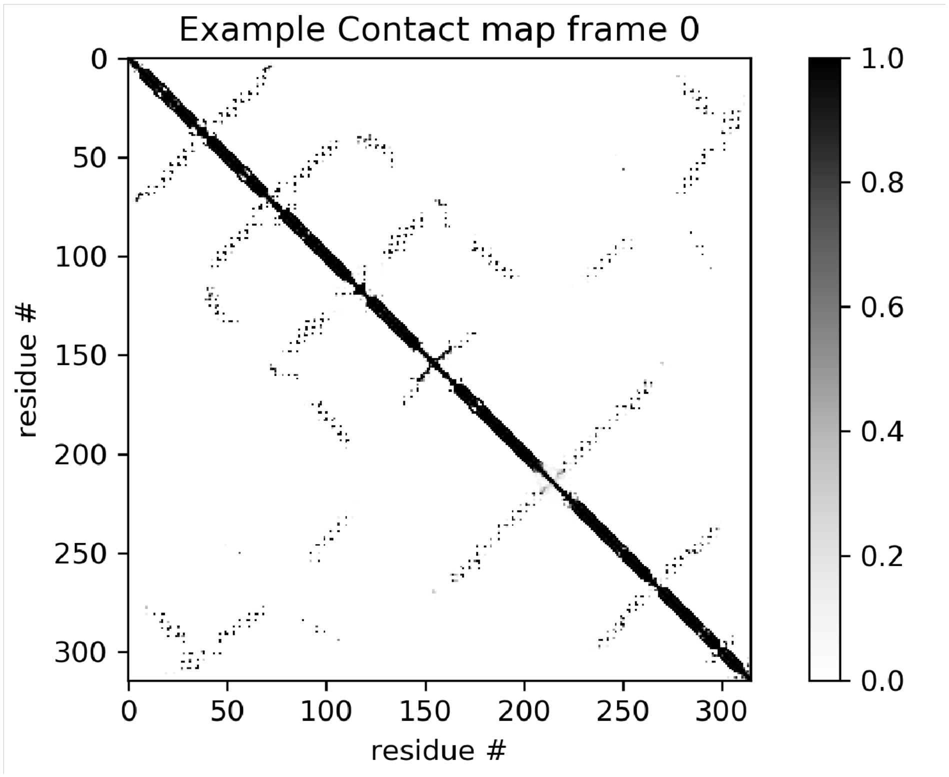

Figure S1: contact map

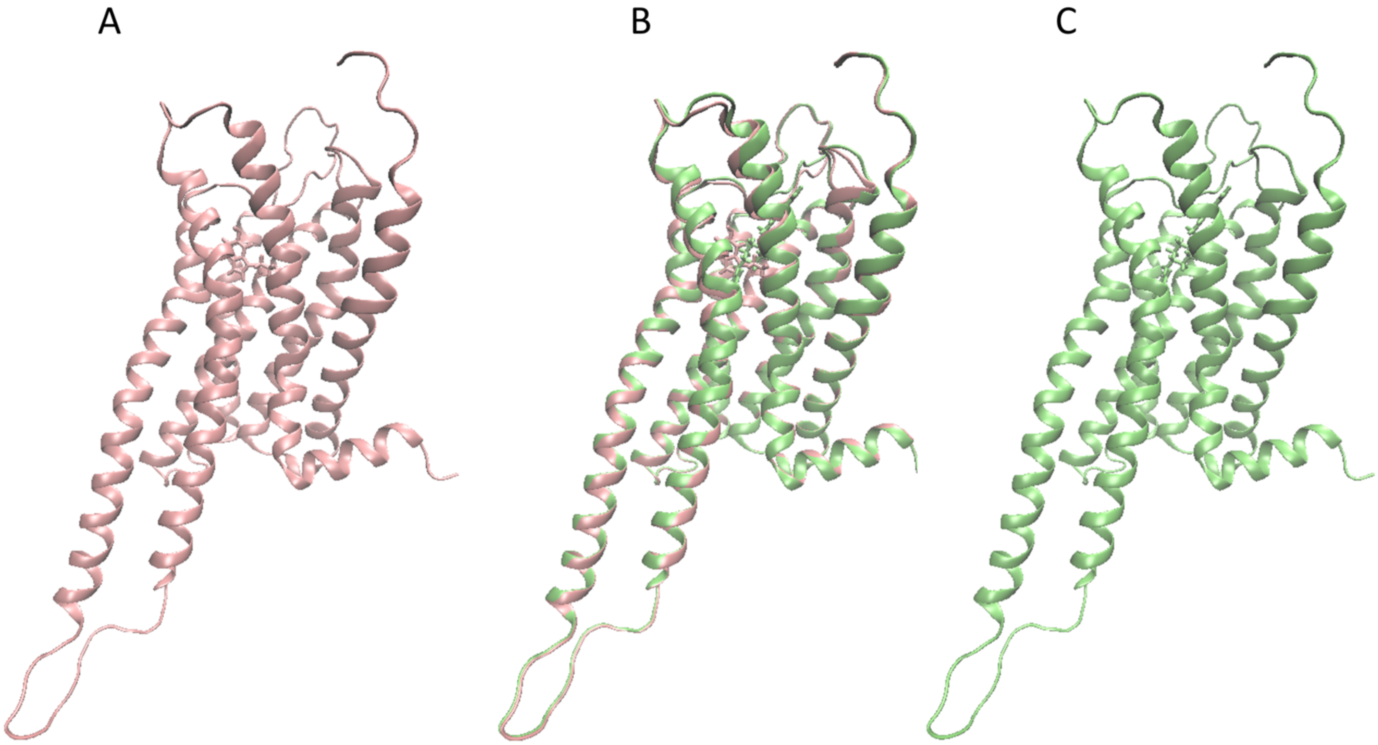

Figure S2: starting structures
Panel A – 5-HT$_{2A}$R/5-HT complex starting structure
Panel C – 5-HT$_{2A}$R/KET complex starting structure
Panel B – 5-HT$_{2A}$R/5-HT and 5-HT$_{2A}$R/KET starting structures overlayed

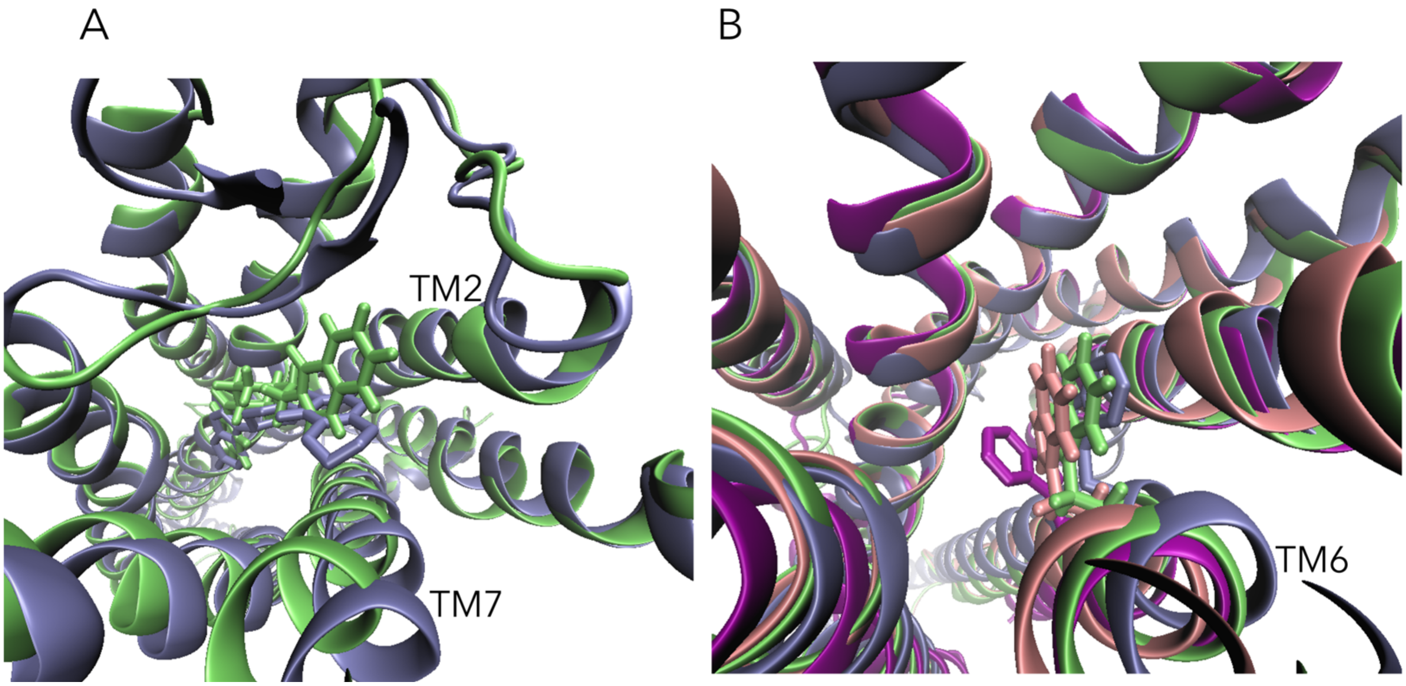

Figure S3: binding pocket
Panel A - 5-HT$_{2A}$R/risperidone complex (blue, pdb 6A93) superposed with 5-HT$_{2A}$R/KET starting structure (green)
Panel B - "toggle switch" W6.48 (shown in stick model) for the 5-HT$_{2A}$R/risperidone (blue), 5-HT$_{2A}$R/25-CN-NBOH/G$_q$ (violet), 5-HT$_{2A}$R/KET (green), and 5-HT$_{2A}$R/5-HT (coral) structures

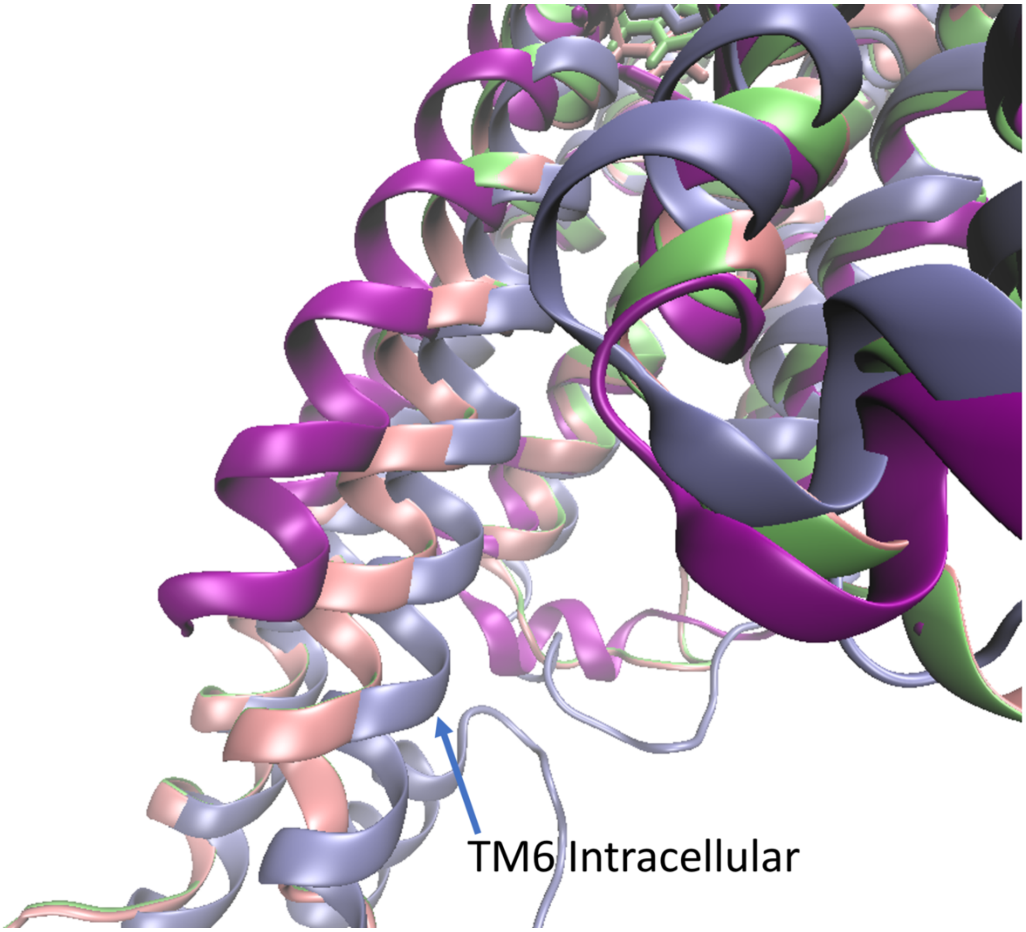

Figure S4: TM6 movements in as observed in inactive and active states of the 5-HT$_{2A}$R 5-HT$_{2A}$R/risperidone (blue), 5-HT$_{2A}$R/KET (green), 5-HT$_{2A}$R/25-CN-NBOH/G$_q$ (violet), and 5-HT$_{2A}$R/5-HT (coral)

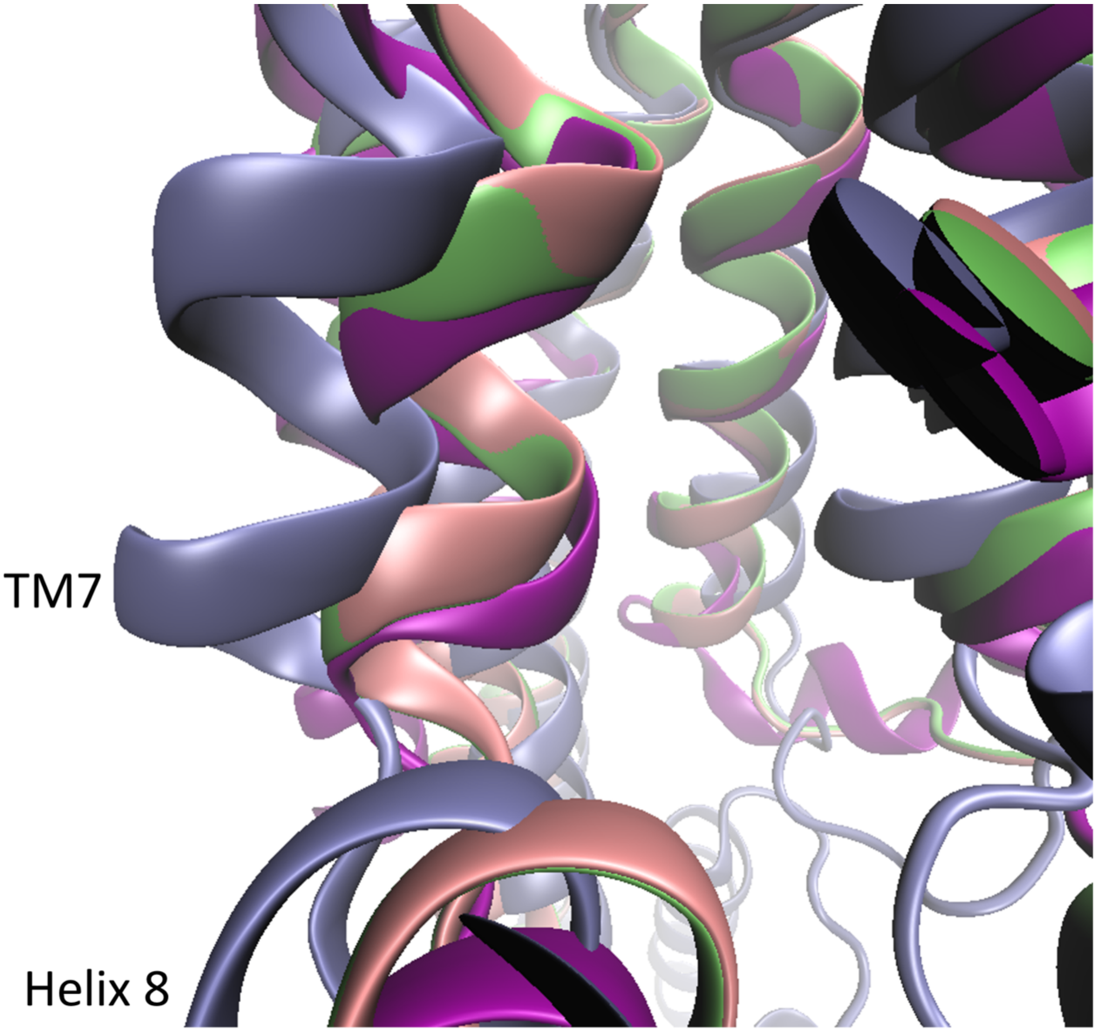

Figure S5: TM7 movements in as observed in inactive and active states of the 5-HT_{2A}R
5-HT_{2A}R/risperidone (blue), 5-HT_{2A}R/25-CN-NBOH/G_q (violet), 5-HT_{2A}R/KET (green), and 5-HT_{2A}R/5-HT (coral)

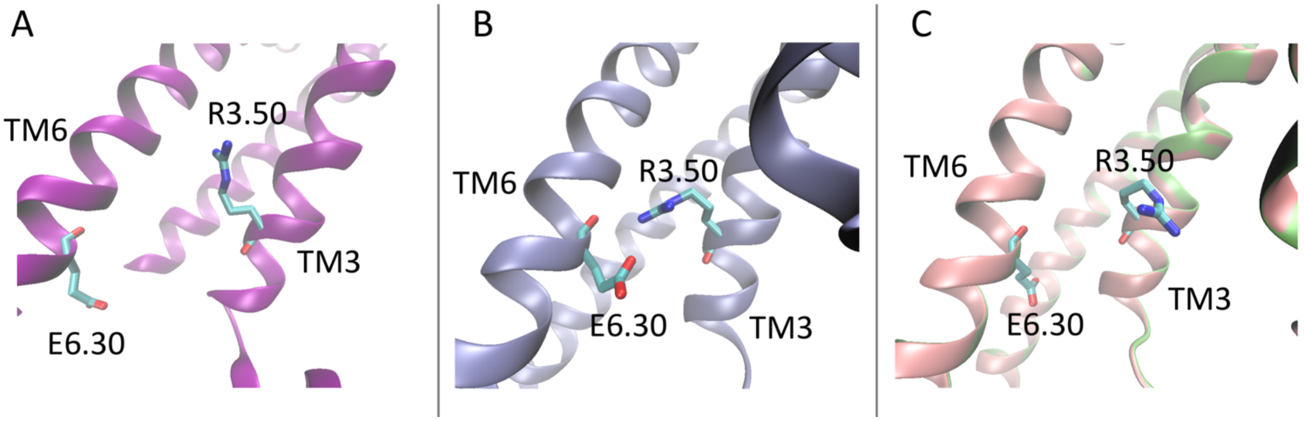

Figure S6: Movements of the ionic lock in inactive and active states of the 5-HT_{2A}R

5-HT_{2A}R/risperidone (blue), 5-HT_{2A}R/25-CN-NBOH/G_q (violet), 5-HT_{2A}R/KET (green), and 5-HT_{2A}R/5-HT (coral)

Panel A - 5-HT_{2A}R/25-CN-NBOH/G_q (violet, pdb 6WHA) with ionic lock (R3.50-E6.30) shown in stick model. Panel B - 5-HT_{2A}R/risperidone (blue), with ionic lock (R3.50-E6.30) shown in stick model. Panel C - 5-HT_{2A}R/KET (green) and 5-HT_{2A}R/5-HT (coral) superposed, with ionic lock (R3.50-E6.30) shown in stick model.

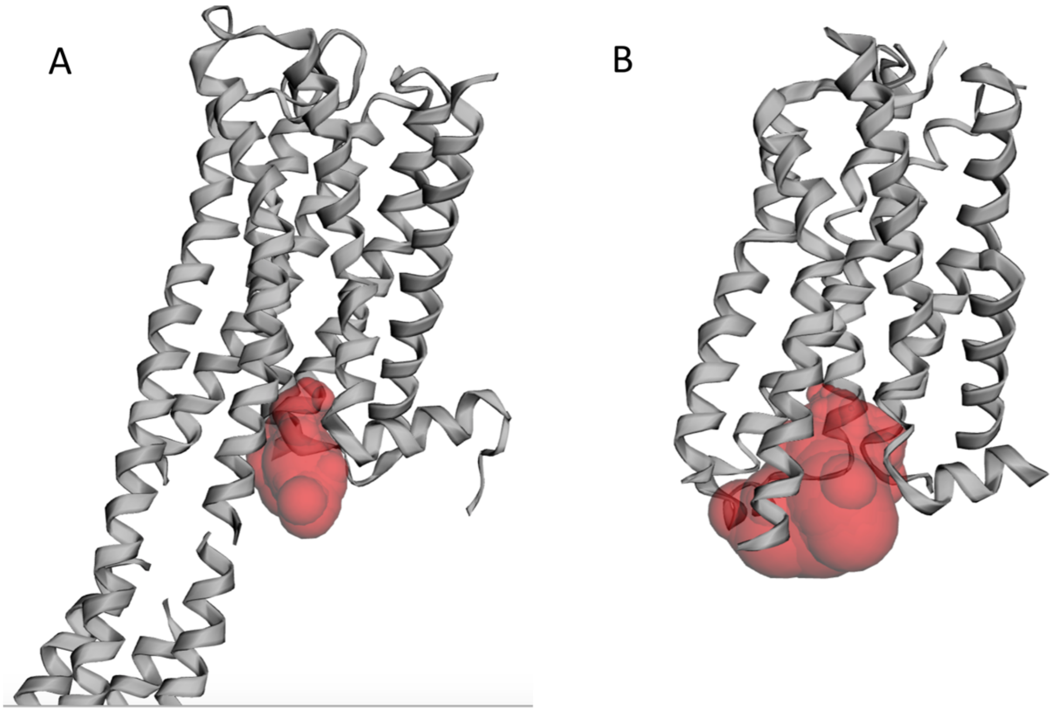

Figure S7: Intracellular cavity volumes (inactive and activated states)
Volume of the intracellular cavity of active and inactive state 5-HT_{2A}R structures. Panel A: 5-HT_{2A}R/risperidone (pdb 6A93) structure is shown in grey, with the intracellular cavity volume computed by the CASTp server shown in red. Panel B: 5-HT_{2A}R/25-CN-NBOH/G_q (violet) (pdb 6WHA) structure is shown in grey, with the intracellular cavity volume computed by the CASTp server shown in red.

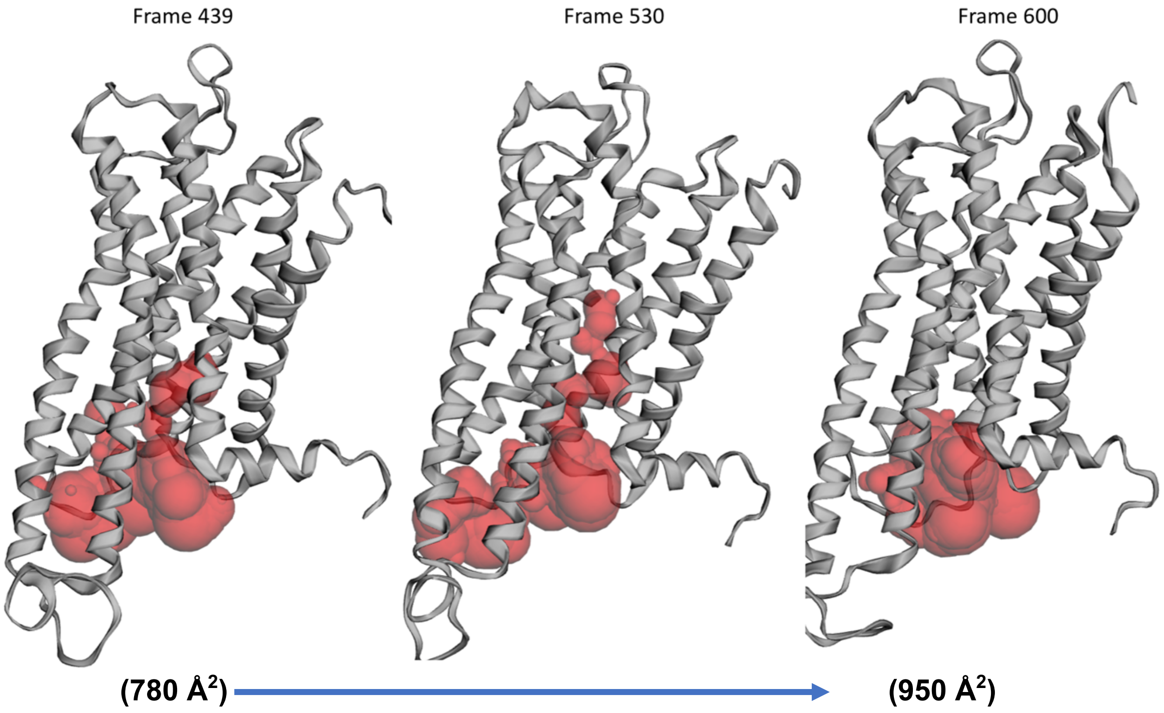

Figure S8: Intracellular cavity volumes changes during activation
Volume of the intracellular cavity, calculated by CASTp webserver, of the 5HT-bound 5-HT$_{2A}$R trajectory at frames corresponding to **Fig1E-G**.

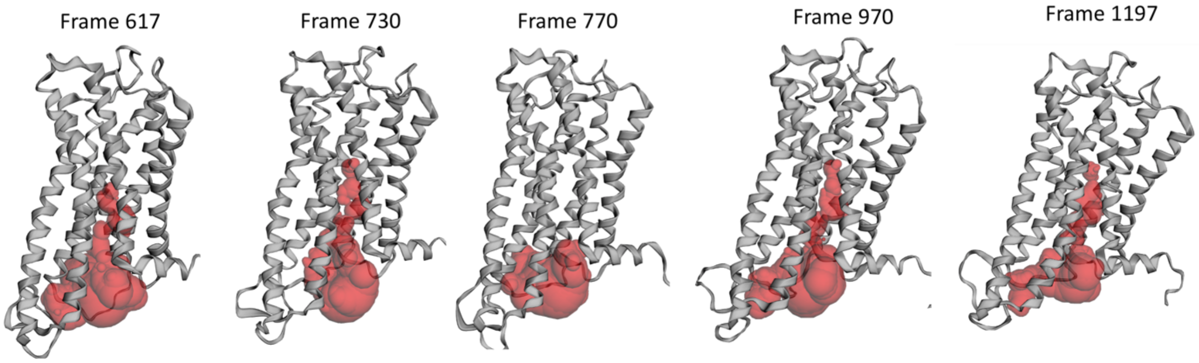

Figure S9: Intracellular cavity volumes changes during inactivation
Volume of the intracellular cavity, calculated by CASTp webserver, of the Ketanserin-bound 5-HT$_{2A}$R trajectory at frames corresponding to **Fig6C-G**.
